# Electrohydrodynamic Drying of Carrot Slices

**DOI:** 10.1371/journal.pone.0124077

**Published:** 2015-04-15

**Authors:** Changjiang Ding, Jun Lu, Zhiqing Song

**Affiliations:** Physical Experiment Center, College of Science, Inner Mongolia University of Technology, Hohhot, China; Sudbury Regional Hospital, CANADA

## Abstract

Carrots have one of the highest levels of carotene, and they are rich in vitamins, fiber and minerals. However, since fresh carrots wilt rapidly after harvest under inappropriate storage conditions, drying has been used to improve their shelf life and retain nutritional quality. Therefore, to further investigate the potential of this method, carrot slices were dried in an EHD system in order to study the effect of different voltages on drying rate. As measures of quality, carotene content and rehydration ratio were, respectively, compared against the conventional oven drying regime. Carotene, the main component of the dried carrot, and rehydration characteristics of the dried product can both indicate quality by physical and chemical changes during the drying process. Mathematical modeling and simulation of drying curves were also performed, using root mean square error, reduced mean square of the deviation and modeling efficiency as the primary criteria to select the equation that best accounts for the variation in the drying curves of the dried samples. Theoretically, the Page model was best suited for describing the drying rate curve of carrot slices at 10kV to 30kV. Experimentally, the drying rate of carrots was notably greater in the EHD system when compared to control, and quality, as determined by carotene content and rehydration ratio, was also improved when compared to oven drying. Therefore, this work presents a facile and effective strategy for experimentally and theoretically determining the drying properties of carrots, and, as a result, it provides deeper insight into the industrial potential of the EHD drying technique.

## Introduction

The consumption of carrots, both fresh and processed, has increased in recent years. This vegetable has one of the highest levels of carotene, and it is rich in vitamins, fiber and minerals. However, fresh carrots wilt rapidly after harvest under unsuitable storage conditions. Drying is an effective method to improve the shelf life and retain the nutritional quality of carrots [1]. Currently, many drying techniques have been applied to carrots, among them solar drying [2], hot air [3], and microwave [4]. Each method has its own set of advantages and disadvantages.

Electrohydrodynamic (EHD) drying is a relatively new and nonthermal drying technique, and many researchers have investigated its use for vegetables, fruits, seafood, grains, and other heat-sensitive materials[5–13]. Compared to hot air drying, these studies found that EHD drying has higher drying rate, consumes less energy and offers superior quality in terms of such physicochemical properties as color, shrinkage, flavor, and nutrient content. At the same time, mathematical modeling and simulation of drying curves under different conditions are important to obtain better control over this process and achieve an overall improvement in the quality of the final product [14]. Many studies have reported the use of mathematical models to describe the EHD drying curves of many products, including, for example, okara cake[15], rough rice[16], fish[17], and mushroom[18], and satisfactory results were obtained. Thus far, however, no studies have reported on the use of EHD for drying carrots. Indeed, the industrial application of EHD is complicated by the fact that no single material has undergone a systematic and comprehensive investigation in relation to EHD processing.

Therefore, this paper reports on the use of EHD for drying carrots, considering drying rate and quality. To accomplish this, carrot slices were dried under different voltages, and five mathematical models were compared to determine which one best represented the drying characteristics of carrots. Effective water diffusion coefficient was measured, as well as carotene content and rehydration ratio of dried carrots. In sum, this work provides a theoretical foundation for establishing control over the drying process from the perspective of the industrial application of EHD across a broad range of fruits and vegetables.

## Materials and Methods

### Experimental Equipment

**Fig 1:**
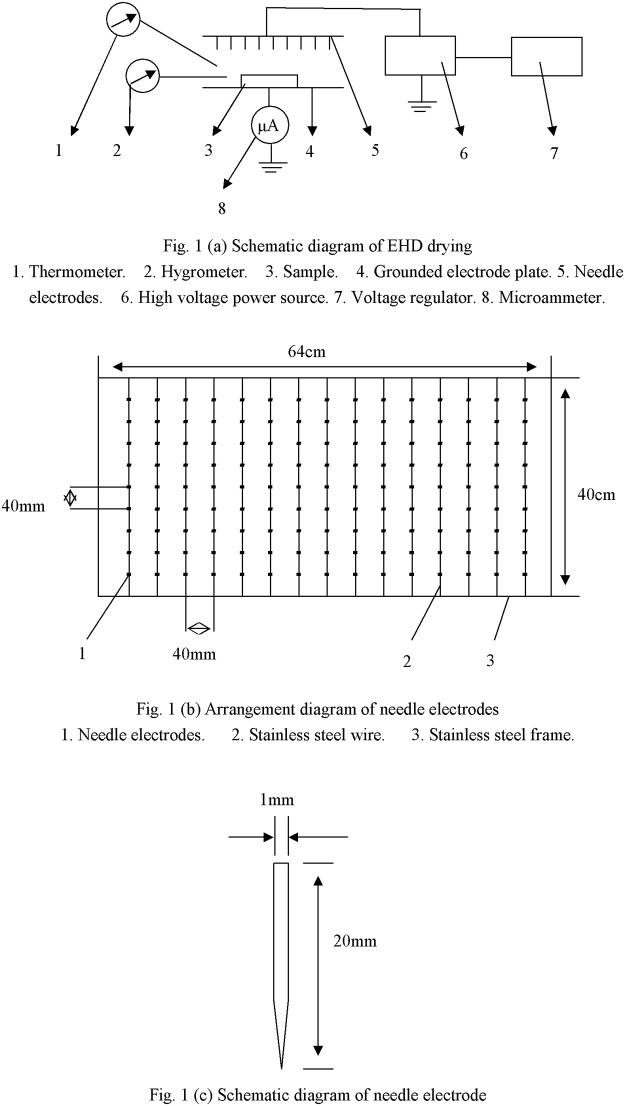
Schematic diagram of EHD drying and needle electrodes.

Fig 1(a) shows a schematic diagram of the experimental setup for EHD drying. It consists of a vertically mounted electrode with multiple sharp pointed needles projected onto a fixed horizontal grounded metallic plate on which the carrot samples to be dried were placed. The distance between the emitting point and the grounded electrode was 100 mm to facilitate the calculation of electric field intensity. The sharp pointed electrodes were connected to a power source able to supply direct current (DC) high voltage or alternating current (AC) high voltage with both positive and negative polarity. The frequency of the electric field was 50Hz. In order to set the desired high voltage parameters for EHD drying, the power was connected to a voltage regulator, with an adjustable voltage ranging from 0–50 kV for alternating current (AC) or 0–70 kV for direct current (DC) by a controller. The grounded plate electrode was an 84cm×44cm rectangular stainless steel plate. Temperature and relative humidity were both measured. Fig 1(b) and 1(c) show the arrangement and schematic diagrams of the needle electrodes, respectively. The needles were 20 mm long with a diameter of 1mm. The distance between two needle electrodes was 40 mm. After some trial-and-error experimentation, this distance was found to be superior to the others based on the correlation between drying rate and energy consumption. The needle electrodes were arranged in multiple rows and lined up by a stainless steel wire. The distance between two stainless steel wires was 40 mm. All the samples were randomly spread in a single layer on the grounded electrode plate.

### Experimental Method

Carrots were purchased from a local market (Inner Mongolia University of Technology, Hohhot, China) and stored in a refrigerator at 4±1°C. The initial moisture content of the carrot was measured by a rapid moisture tester (Sh10A, Shanghai Luheng Instrument Co., Ltd., Shanghai, China). The initial moisture content was 90±1%. The drying temperature was 21±2°C, the drying relative humidity was 30±5%, and the ambient wind speed was 0m/s.

Moisture content and moisture ratio in the drying process is defined as

$$m_{g}=m_{0}\times (1-M_{0})$$ (1)

$$M_{i}=\frac{m_{i}-m_{g}}{m_{g}}\times 100\%$$ (2)

$$MR=\frac{M_{i}-M_{e}}{M_{0}-M_{e}},$$ (3)

where *M*_0_ is the initial moisture content of carrot, *M*_*e*_ is the equilibrium moisture content of carrot, *M*_*i*_ is the moisture content of carrot (gwatergdry  matter) at time *t*_*i*_, *m*_0_ is the initial mass of carrot, *m*_*i*_ is the mass of carrot at time *t*_*i*_, and *m*_*g*_ is the dry matter of carrot.

Drying rate was defined as the change in the mass per unit area of samples and per unit time (kg water/ m^2^h). To quantify it, the following equation was used:

$$v_{i}=\frac{m_{i-1}-m_{i}}{S(t_{i}-t_{i-1})}$$ (4)

where *S* is the cross-sectional area of carrot slice.

Before the experiments, the carrots were removed from the refrigerator and brought to room temperature naturally. They were washed, peeled and cut into rectangular slices 13 mm in length, 8 mm in width and 1 mm in thickness for experiments, changing voltage each time at 0kV, 5kV, 10kV, 15kV, 20kV, 25kV and 30kV for AC electric field, respectively. The electric current was measured by a microammeter.

The mass of sample in the drying process was measured by a Sartorius Analytical Balance BS124S (Goettingen, Germany) every 0.5 hour. The moisture ratio and drying rate were calculated using Eqs (3) and (4).

### Mathematical Model

For mathematical modeling, five different models were applied to find the model best suited for describing the drying rate curve of carrot slices at 10kV, 15kV, 20kV, 25kV and 30kV (Table 1).

**Table 1:** Mathematical models applied to the drying curves.

Model name	Model equation	References
Lewis	*MR* = *e* ^*-kt*^	[18]
Page	$MR=e^{-kt^{n}}$	[15]
Henderson and Pabis	*MR* = *αe* ^*-k*^	[14,19]
Logarithmic	*MR* = *αe* ^*-k*^ + *b*	[14,19]
Quadratic	*MR* = *α + bt + ct* ^*2*^	[17]

### Statistical Parameters

The parameters of the mathematical model were estimated by nonlinear regression. Some statistical parameters were used to evaluate the goodness of model fit. The root mean square error (*E*_*RMS*_), reduced mean square of the deviation (*χ* ^2^) and modeling efficiency (*EF*) were used as the primary criteria to select the equation that best accounts for the variation in the drying curves of the dried samples [14,19,20]. *χ* ^2^ was used to determine the goodness of the fit: the lower the values of *χ* ^2^, the better the goodness of the fit. *E*_*RMS*_ gives the deviation between the predicted and experimental values. *EF* also gives predictive power of the model relative to the drying behavior of the product, and its highest value is 1. These statistical values can be calculated as follows:

$$E_{RMS}=\sqrt{\frac{1}{N}\sum_{i=1}^{N}{\left(MR_{pre,i}-MR_{exp,i}\right)}^{2}}$$ (5)

$$\chi^{2}=\frac{\sum_{i=1}^{N}{\left(MR_{\exp,i}-MR_{pre,i}\right)}^{2}}{N-n}$$ (6)

$$EF=\frac{\sum_{i=1}^{N}{\left(MR_{\exp,i}-MR_{\exp mean}\right)}^{2}-\sum_{i=1}^{N}{\left(MR_{pre,i}-MR_{\exp,i}\right)}^{2}}{\sum_{i=1}^{N}{\left(MR_{\exp,i}-MR_{\exp mean}\right)}^{2}},$$ (7)

where *MR*_exp, *i*_ is the *i*th experimental moisture, *MR*_*pre*, *i*_ is the *i*th predicted moisture, *N* is the number of observations, *n* is the number of constants in the drying model, and *MR*_exp*mean*_ is the mean value of experimental moisture.

### Effective water diffusion coefficient (*D*_*eff*_)

Assuming the main mechanism of drying as being diffusive in nature, experimental results can be interpreted by considering Fick’s second diffusion equation:

$$\frac{dM}{dt}=D_{eff}\frac{d^{2}M}{dr^{2}}$$ (8)

If the moisture movement by thermal gradient within the thin slice is ignored, moisture transfer can be considered as a one-dimensional diffusion process in the upward direction from the bottom of the sample towards the top surface. In this diffusion analysis, it was also assumed that (1) the initial moisture content of sample is constant; (2) the moisture movement follows a diffusive pattern; (3) shrinkage is negligible; and (4) diffusion coefficients are constant for each voltage.

For long drying periods, when *L* is small and *t* is large, limiting forms of equations are obtained for the slice, and the equation can be expressed as[21–23]

$$MR=\frac{8}{\pi^{2}}\cdot exp(-\frac{\pi^{2}D_{eff}t}{4L^{2}}),$$ (9)

where *L* is half of the thickness of carrot slice (m). This equation can be written as

$$ln[MR]=-\frac{\pi^{2}D_{eff}}{4L^{2}}t+ln[\frac{8}{\pi^{2}}].$$ (10)

*D*_*eff*_ can be measured from the plot of ln[*MR*] against time. The diffusion coefficient for each voltage was calculated by substituting the experimental data in the previous equation. In practice, such coefficient was determined by plotting experimental data in terms of ln[*MR*] versus drying time. From Eq (11), a plot of ln[*MR*]versus drying time gives a straight line with slope:

$$\text{slope}=-\frac{\pi^{2}D_{eff}}{4L^{2}}.$$ (11)

The use of Eq (12) is based on the assumption of constant moisture diffusivity for each voltage, which predicts a linear behavior for the dependence of ln[*MR*] versus drying time.

### The control experiment between EHD and oven drying

In previous studies [24], it was found that the drying rate in an EHD system increases with the increase of voltage. To detect the effective constituent of dried samples under higher voltage, including EHD combined with hot drying, we performed the drying experiment at 35kV and 40°C, using conventional oven drying as control. Carrots weighing a total of 1,858 kg were used to conduct the experiment. The carrots were cut into rectangular strips 5 mm in length, 3 mm in width and 0.5 mm in thickness and equally divided into 2 groups. One group was subjected to EHD drying with 35 kV at experimental temperature (40±1°C) (combination of EHD and hot drying) and relative humidity (30±5%) in the EHD drying equipment (GXJ-2, designed by Inner Mongolia University, Hohhot, China). Another group was oven dried at 70°C, the temperature of industrial drying, using a model DGX-9053B-1 oven (Shanghai Fuma Laboratory Instrument Co., Ltd., Shanghai, China). The oven’s internal dimensions were 42cm×35cm×40cm. Carrot strips were paved to 5mm in thickness for both EHD and oven drying. The carotene content and rehydration ratio of dried carrot slices were determined to analyze the quality.

### Determination of Carotenes

Carotenes were extracted according to the method described by Zhang et al. [25] with some modifications. 3g of crushed samples and 30 mL of petroleum ether with a boiling range between 60 and 90°C were placed into a round bottom flask. The mixture was condensed and reflowed for 40min at 60°C, followed by filtration. The filtrate was constant volume to 25mL by petroleum ether. The light absorption values were measured at 450nm. The carotene content was measured by the linear regression equation. Experiments were independently performed three times in this study and an average taken.

### Rehydration Ratio

To evaluate the rehydration ratio (RR) of dried samples, 12 g of dried carrot slices were immersed in 144 mL of distilled water at ambient conditions for 30min. Then the carrot slices and distilled water were boiled for 5min. The carrot slices were weighed after removing excess water with the help of absorbent paper. RR was determined using the following equation:

$$RR=\frac{m_{a}}{m_{b}},$$ (12)

where *m*_*b*_ and *m*_*α*_ are weights of the samples before and after rehydration, respectively. Experiments were independently performed three times in this study and an average taken.

### Statistical Analysis

SPSS Version 16.0 for Windows was used for statistical analysis, and one-way ANOVA (single-factor analysis of variance) was used to calculate the moisture ratio between the carrot slices under alternating electric field and without electric field (control). The differences in moisture ratio are considered statistically significant when *p*<0.05 The experiments were done at 6 voltages from 5kV to 30kV, and each was compared with the control group (without electric field), respectively.

Carotene concentrations and rehydration ratios were also calculated between the carrot slices treated under EHD and oven drying using single-factor analysis of variance. The results reported in this study are presented as means ± standard deviation (SD).

## Results and Discussion

**Fig 2:**
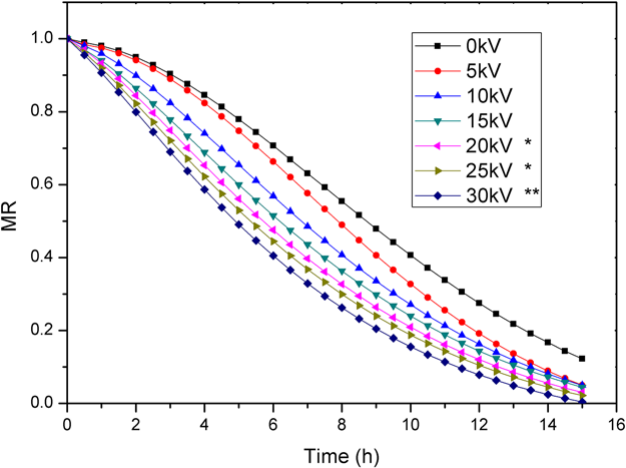
Moisture ratio of carrots under different voltages. *p<0.05, significant difference; **p<0.01, very significant difference.

**Fig 3:**
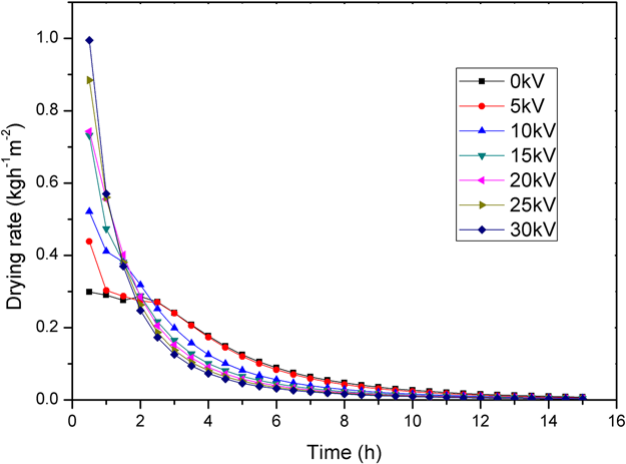
Drying rate of carrots under different voltages.

The moisture ratio and drying rate curve of carrot samples treated with different voltage values are shown in Figs 2 and 3, respectively.

It is clear that the moisture ratio of carrot samples was significantly reduced under EHD compared with that of control. We calculated the moisture ratio for carrot slices under alternating electric field and without electric field using single-factor analysis of variance. Compared to control, moisture ratio values showed statistically significant difference at 20kV and 25kV (p<0.05) and very statistically significant difference at 30kV (p<0.01), whereas no statistically significant difference was observed at 5, 10 and 15kV (p>0.05). The drying rate decreased with the decrease of moisture ratio. The drying rate of carrot samples treated with EHD increased compared to that of the control, and increasing the voltage had a major effect on the enhancement of the drying rate, increasing by 0.47, 0.75, 1.45, 1.49, 1.96 and 2.33 times, respectively, at 5, 10, 15, 20, 25 and 30 kV voltages compared to that of the control in the first half hour. Compared to control, the application of EHD contributed toward a decrease in the drying time by 3.3%, 6.7%, 13.3%, 20.0%, 23.3% and 26.7%, respectively, to reach a final moisture content of 17% at 5, 10, 15, 20, 25 and 30 kV. These results agree with those studies which reported enhancement in drying rate with increase of applied voltage [16,26–28]. The electric current is 0μA, 0μA, 1.3μA, 4.1μA, 6.5μA and 7.5μA, respectively, at 5, 10, 15, 20, 25 and 30 kV voltages. This indicates the absence of corona wind in the 5 and 10kV. Higher voltage or electric field strength can induce stronger ionic wind and higher wind velocity [29]. The enhancement in mass transfer rate could be attributed to the corona wind [11]. The corona wind produced impinges on the moist material and disturbs the saturated air layer, leading to evaporation enhancement[12]. The pressure of ionic wind centralizes at the central part, while the pressure is small at edges [29]. Therefore, placement of samples directly below the needle point electrodes may have largely improved drying rate. The enhancement in drying rate by the multiple points-to-plate could be attributed to electric wind created by each needle point electrode, resulting in a cumulative effect that could have greatly increased the drying rate[17]. Still, apart from corona wind under the AC electric field, another EHD drying mechanism is possible. Specifically, water molecules are highly polar molecule structures and orient themselves in the direction of the electric field, which would lead to the conversion of electrical energy into mechanical energy, thereby forcing water molecules out of the material under AC electric field. This effect would be directly proportional to the electric field strength.

The constants and coefficients of five mathematical models at different drying conditions are given in Table 2.

**Table 2:** Results of statistical analyses on the modeling of moisture ratio and drying time.

Model	Voltage	*n*	*k*	*a*	*b*	*c*	*E*_*RMS*_	*χ* ^2^	*EF*
Lewis	10kV		0.1169				0.083224	0.007157	0.928333
	15kV		0.1287				0.067934	0.004769	0.950555
	20kV		0.1398				0.063217	0.004130	0.957811
	25kV		0.1493				0.057563	0.003424	0.964794
	30kV		0.1635				0.055645	0.003200	0.967720
Page	10kV	1.6568	0.0294				0.009922	0.000102	0.998981
	15kV	1.5009	0.0459				0.010705	0.000118	0.998772
	20kV	1.4523	0.0562				0.010911	0.000123	0.998743
	25kV	1.4003	0.0676				0.012061	0.000150	0.998454
	30kV	1.3791	0.0788				0.014127	0.000206	0.997919
Henderson and Pabis	10kV		0.1355	1.1417			0.064598	0.004312	0.956822
	15kV		0.1451	1.1180			0.053012	0.002904	0.969890
	20kV		0.1563	1.1129			0.049214	0.002503	0.974431
	25kV		0.1649	1.1024			0.045475	0.002137	0.978027
	30kV		0.1795	1.0985			0.044798	0.002074	0.979078
Logarithmic	10kV		0.0408	2.2771	-1.2205		0.021721	0.000488	0.995118
	15kV		0.0646	1.6579	-0.6119		0.017195	0.000306	0.996832
	20kV		0.0816	1.4739	-0.4275		0.016005	0.000265	0.997296
	25kV		0.0948	1.3715	-0.3307		0.014127	0.000206	0.997880
	30kV		0.1103	1.3043	-0.2654		0.013466	0.000187	0.998110
Quadratic	10kV			1.0579	-0.0921	0.0015	0.020337	0.000427	0.995721
	15kV			1.0437	-0.1024	0.0023	0.013988	0.000202	0.997904
	20kV			1.0401	-0.1114	0.0029	0.011234	0.000130	0.998668
	25kV			1.0301	-0.1171	0.0033	0.008224	0.000070	0.999281
	30kV			1.0227	-0.1252	0.0038	0.006635	0.000045	0.999541

The values of the root mean square error (*E*_*RMS*_), reduced mean square of the deviation (*χ* ^2^) and modeling efficiency (*EF*) are also given in Table 2. In all cases, the *EF* values for the models were greater than 0.92, indicating a good fit. It is clear that all five mathematical models could satisfactorily describe drying curves of carrot slices dried under EHD. The results also indicated that the highest values of *EF* and the lowest values of *E*_*RMS*_ and *χ* ^2^could be obtained by the Page model. Consequently, the Page model was determined to be the best for describing the drying characteristics of carrot slices. From Table 2, the values of *E*_*RMS*_, *χ* ^2^, and *EF* for the Page model vary between 0.009922 and 0.014127, 0.000102 and 0.000206, and 0.997919 and 0.998981, respectively. We also found that *k* values for the Lewis, Page, Henderson and Pabis, and Logarithmic models increased as voltage increased and that the *n* values for the Page model and the *a* values for the Henderson and Pabis, Logarithmic and Quadratic models decreased as voltage increased. Since the drying process itself is concerned with the texture and geometry of the materials, as well as the drying technology, wide differences can be observed for different materials with same drying technique. It is equally difficult to describe the drying process; therefore, it can be expected that the mathematical description of it would be correspondingly difficult. Nonetheless, this paper also aimed to find a mathematical model that best describes carrot drying under EHD treatment. To accomplish this, five different models were compared (see Table 1), and based on the results discussed above, the Page model was determined to be the best for describing the drying characteristics of carrot slices between control and EHD.

Using the linear fit function of Origin 8.0, the following equations between ln[*MR*] and drying time are given for each voltage:

0kV ln[MR]=-0.1354t+0.3141   R2=0.93015kVln[MR]=-0.1818t+0.4597   R2=0.900310kV ln[MR]=-0.1882t+0.3874   R2=0.939015kV ln[MR]=-0.1956t+0.3520   R2=0.951720kV ln[MR]=-0.2146t+0.3789   R2=0.948825kV ln[MR]=-0.2302t+0.3975   R2=0.944130kV ln[MR]=-0.2819t+0.5587   R2=0.9024

Even though the experimental drying dependence is not strictly linear in a logarithmic scale, a linear fit appears to be a quite successful approximation because the coefficients of determination *R* ^2^ are always greater than 0.90 for each voltage. Therefore, the assumption of constant value of diffusion moisture for each voltage works reasonably well. As a consequence, such slope can be regarded as a useful approximation for the value of diffusivity.

The effective diffusion coefficient (*D*_*eff*_) values under different voltages can be seen in Table 3. The *D*_*eff*_ values ranged approximately from 1.37 ×10^-8^m^2^/s to 2.86 × 10^-8^m^2^/s under different voltages. The results show that *D*_*eff*_ values increased with increasing voltage, in turn indicating that *D*_*eff*_ values would affect drying rate. Dinani et al. [30] reported that voltage in combined EHD/hot air drying significantly affects *D*_*eff*_ and that the internal diffusion of water in material plays an important role in EHD drying. These conclusions are corroborated in the present study.

**Table 3:** Effective diffusion coefficient (*Def*_*f*_) values under different voltages.

Voltage	30kV	25 kV	20 kV	15 kV	10 kV	5 kV	0 kV
*D*_*eff*_(×10^-8^ m^2^/s)	2.86	2.33	2.17	1.98	1.91	1.84	1.37

The experimental data for carrots dried under two different methods can be seen in Table 4. The results indicate that combined EHD/oven drying can significantly reduce the drying time for carrots.

**Table 4:** Experimental data of carrots submitted to two drying methods.

Drying methods	Mass of carrot (kg)	Thickness of carrot strips(mm)	Drying time (min)	Drying temperature (°C)	Voltage (kV)	Initial moisture content (%)	Final moisture content (%)
EHD drying	0.929	5	280	40	35	91	6
Oven drying	0.929	5	430	70	-	91	6

Carotene, the main component of the dried carrot, was used as a quality index because it can indicate physical and chemical changes in the samples during drying. The carotene contents of EHD and oven-dried carrot slices are given in Table 5. It can be seen that the application of EHD contributed toward an increase by 11.53% in the carotene contents of dried carrots, compared to oven drying.

**Table 5:** Carotene contents of EHD and oven-dried carrot slices.

Test sample	EHD drying	Oven drying
Carotene contents (mg/(100g))	43.5±0.3*	39.0±0.4*

*p<0.05, significant difference.

The rehydration characteristics of the dried product can be a good indicator of the physical and chemical changes of samples during drying and can be widely used as an index for evaluating dried sample quality. As shown in Table 6, the application of EHD contributed toward an increase by 11.65% in the rehydration ratio of dried carrots, compared to oven drying, indicating that the dried product treated by EHD has higher rehydration capacity.

**Table 6:** Rehydration ratio of EHD and oven-dried carrot slices.

Test sample	EHD drying	Oven drying
Rehydration ratio	8.24±0.20*	7.38±0.03*

*p<0.05, significant difference

## Conclusion

The electrohydrodynamic (EHD) technique may enhance the drying rate of carrots. The drying rate under EHD increases with increasing voltage. Based on the root mean square error (*E*_*RMS*_), reduced mean square of the deviation (*χ* ^2^) and modeling efficiency (*EF*), the Page model was found to be suitable for describing the drying characteristics of carrot slices under different voltages. Voltage has a major effect on *D*_*eff*_ values. Carrot samples dried by EHD had higher carotene content and rehydration ratio than samples submitted to oven drying. Taken together, it can be concluded that EHD achieves better quality than oven drying for this vegetable.

## References

[1] Wu B, Ma H, Qu W, Wang B, Zhang X, Wang P, et al Catalytic infrared and hot air dehydration of carrot slices. J Food Process Eng. 2014; 37: 111–121.

[2] Romanoa G, Kocsisb L, Farkasc I. Analysis of energy and environmental parameters during solar cabinet drying of apple and carrot. Drying Technol. 2009; 27: 574–579.

[3] Srikiatden J, Roberts JS. Predicting moisture profiles in potato and carrot during convective hot air drying using isothermally measured effective diffusivity. J Food Eng. 2008; 84: 516–525.

[4] Li Z, Raghavan GSV, Wang N. Carrot volatiles monitoring and control in microwave drying. LWT-Food Sci Technol. 2010; 43: 291–297.

[5] Esehaghbeygi A. Effect of electrohydrodynamic and batch drying on rice fissuring. Drying Technol. 2012; 30: 1644–1648.

[6] Bai YX, Yang GJ, Hu YC, Qu M. Physical and sensory properties of electrohydrodynamic (EHD) dried scallop muscle. J Aquat Food Prod T. 2012; 21: 238–247.

[7] Alemrajabi AA, Rezaee F, Mirhosseini M, Esehaghbeygi A. Comparative evaluation of the effects of electrohydrodynamic, oven, and ambient air on carrot cylindrical slices during drying process. Drying Technol. 2012; 30: 88–96.

[8] Esehaghbeygi A, Basiry M. Electrohydrodynamic (EHD) drying of tomato slices (*Lycopersicon esculentum*). J Food Eng. 2011; 104: 628–631.

[9] Bai YX, Sun B. Study of electrohydrodynamic (EHD) drying technique for shrimps. J Food Process Pres. 2011; 35: 891–897.

[10] Basiry M, Esehaghbeygi A. Electrohydrodynamic (EHD) drying of rapeseed (*Brassica napus* L.). J Electrostat. 2010; 68: 360–363.

[11] Hashinaga F, Bajgai T, Isobe S, Barthakur N. Electrohydrodynamic (EHD) drying of apple slices. Drying Technol. 1999; 17: 479–495.

[12] Singh A, Orsat V, Raghavan V. A comprehensive review on electrohydrodynamic drying and high-voltage electric field in the context of food and bioprocessing. Drying Technol. 2012; 30: 1812–1820.

[13] Bai Y, Qu M, Luan Z, Li X, Yang Y. Electrohydrodynamic drying of sea cucumber (*Stichopus japonicus*). LWT-Food Sci Technol. 2013; 54: 570–576.

[14] Meisami-asl E, Rafiee S, Keyhani A, Tabatabaeefar A. Mathematical modeling of moisture content of apple slices (var. Golab) during drying. Pak J Nutr. 2009; 8: 804–809.

[15] Li F, Li L, Sun J, Tatsumi E. Electrohydrodynamic (EHD) drying characteristic of okara cake. Drying Technol. 2005; 23: 565–580.

[16] Cao W, Nishiyama Y, Koide S, Lu Z. Drying enhancement of rough rice by an electric field. Biosyst Eng. 2004; 87: 445–451.

[17] Bai YX, Li XJ, Sun Y, Shi H. Thin layer electrohydrodynamic (EHD) drying and mathematical modeling of fish. Int J Appl Electrom Mech. 2011; 36: 217–228.

[18] Dinani ST, Hamdami N, Shahedi M, Havet M. Mathematical modeling of hot air/electrohydrodynamic (EHD) drying kinetics of mushroom slices. Energ Convers Manage. 2014; 86: 70–80.

[19] Menges HO, Ertekin C. Mathematical modeling of thin layer drying of Golden apples. J Food Eng. 2006; 77: 119–125.

[20] Demir V, Gunhan T, Yagcioglu A. Mathematical modelling of convection drying of green table olives. Biosyst Eng. 2007; 98: 47–53.

[21] Pardeshi I, Chattopadhyay P. Hot air puffing kinetics for soy-fortified wheat-based ready-to-eat (RTE) snacks. Food Bioprocess Tech. 2010; 3: 415–426.

[22] Ruiz Celma A, Rojas S, Lopez-Rodriguez F. Mathematical modelling of thin-layer infrared drying of wet olive husk. Chem Eng Process. 2008; 47: 1810–1818.

[23] Senadeera W, Bhandari BR, Young G, Wijesinghe B. Influence of shapes of selected vegetable materials on drying kinetics during fluidized bed drying. J Food Eng. 2003; 58: 277–283.

[24] Ding C, Lu J, Song Z, Bao S. The drying efficiency of electrohydrodynamic (EHD) systems based on the drying characteristics of cooked beef and mathematical modeling. Int J Appl Electrom Mech. 2014; 46: 455–461.

[25] Zhang J, Zhang Z, Wu Y. Method of determination of the content of β-carotene in carrot. J Inner Mongolia Agricul Univ. 2000; 21: 121–124.

[26] Li F-D, Li L-T, Sun J-F, Tatsumi E. Effect of electrohydrodynamic (EHD) technique on drying process and appearance of okara cake. J Food Eng. 2006; 77: 275–280.

[27] Lai F, Lai K-W. EHD-enhanced drying with wire electrode. Drying Technol. 2002; 20: 1393–1405.

[28] Balcer B, Lai F. EHD-enhanced drying with multiple-wire electrode. Drying Technol. 2004; 22: 821–836.

[29] Zhang Y, Liu L, Ouyang J. On the negative corona and ionic wind over water electrode surface. J Electrostat. 2014; 72: 76–81.

[30] Dinani ST, Havet M, Hamdami N, Shahedi M. Drying of mushroom slices using hot air combined with an electrohydrodynamic (EHD) drying system. Drying Technol. 2014; 32: 597–605.

